# Fate and Persistence of a Pathogenic NDM-1-Positive Escherichia coli Strain in Anaerobic and Aerobic Sludge Microcosms

**DOI:** 10.1128/AEM.00640-17

**Published:** 2017-06-16

**Authors:** David Mantilla-Calderon, Pei-Ying Hong

**Affiliations:** King Abdullah University of Science and Technology (KAUST), Water Desalination and Reuse Center (WDRC), Biological and Environmental Science and Engineering Division (BESE), Thuwal, Saudi Arabia; Shanghai Jiao Tong University

**Keywords:** persister cells, bacterial decay, extracellular DNA decay, antibiotic resistance genes, horizontal gene transfer, wastewater treatment

## Abstract

The presence of emerging biological pollutants in treated wastewater effluents has gained attention due to increased interest in water reuse. To evaluate the effectiveness of the removal of such contaminants by the conventional wastewater treatment process, the fate and decay kinetics of NDM-1-positive Escherichia coli strain PI7 and its plasmid-encoded antibiotic resistance genes (ARGs) were assessed in microcosms of anaerobic and aerobic sludge. Results showed that E. coli PI7 decayed at a significantly lower rate under anaerobic conditions. Approximate half-lives were 32.4 ± 1.4 h and 5.9 ± 0.9 h in the anaerobic and aerobic microcosms, respectively. In the aerobic microcosms, after 72 h of operation, E. coli PI7 remained detectable, but no further decay was observed. Instead, 1 in every 10,000 E. coli cells was identified to be recalcitrant to decay and persist indefinitely in the sludge. ARGs associated with the E. coli PI7 strain were detected to have transferred to other native microorganisms in the sludge or were released to the liquid fraction upon host decay. Extracellular DNA quickly degraded in the liquid fraction of the aerobic sludge. In contrast, no DNA decay was detected in the anaerobic sludge water matrix throughout the 24-h sampling period. This study suggests an increased likelihood of environmental dispersion of ARGs associated with anaerobically treated wastewater effluents and highlights the potential importance of persister cells in the dissemination of E. coli in the environment during reuse events of treated wastewater.

**IMPORTANCE** This study examines the decay kinetics of a pathogenic and antibiotic resistant strain of Escherichia coli in microcosms simulating biological treatment units of aerobic and anaerobic sludge. The results of this study point at a significantly prolonged persistence of the E. coli and the associated antibiotic resistance gene in the anaerobic sludge. However, horizontal transfer of the plasmid encoding the antibiotic resistance gene was detected in the aerobic sludge by a cultivation method. A subpopulation of persister E. coli cells was also detected in the aerobic sludge. The findings of this study suggest potential areas of concern arising from pathogenic and antibiotic-resistant E. coli during both anaerobic and aerobic sludge treatment processes.

## INTRODUCTION

In most wastewater treatment plants (WWTPs), aerobic activated sludge processes are conventionally used as the main biological unit to achieve removal of organic materials from wastewater but such systems incur a large energy cost (1). Anaerobic digesters are increasingly being considered for use as an alternative process for wastewater treatment due to their various advantages (2). Energy can be recovered from the wastewater in the form of methane produced from anaerobic fermentation of the organic carbon, and the amount of sludge produced from anaerobic processes is lower than that from aerobic processes (1).

However, municipal wastewater often contains subtherapeutic levels of antibiotic residues, and the continuous exposure to antibiotics can select for antibiotic-resistant bacteria (ARB) within both aerobic and anaerobic sludge processes. In recent years, WWTPs had been shown to be potential hot spots for ARB and antibiotic resistance gene (ARG) propagation (3). Despite having undergone treatment, the treated municipal wastewater can still contain significant amounts of ARB and ARGs. For example, an earlier study showed that treated effluents that ultimately would be intended for reuse or discharged into receiving water bodies carried about 10^6^ to 10^11^ heterotrophic CFU per cubic meter (4, 5), from which 16 to 28% corresponded to ARB (5). In another study, 10^7^ to 10^9^ copies of diverse tetracycline resistance genes were found in each cubic meter of chlorinated effluents (4). To further compound this problem, genes that confer resistance to carbapenems (i.e., *bla*_NDM-1_), which are antibiotics used as a last line of defense against multidrug-resistant infections (6, 7), were also detected at alarming levels in final effluents, approaching 10^9^ copies per cubic meter of treated wastewater (8).

This problem is of particular concern in water-scarce countries with pressing needs to reuse the treated wastewater. The incidence of bacterial pathogens carrying ARGs that confer resistance to antibiotics of last resort (e.g., *bla*_NDM-1_-positive pathogenic Escherichia coli) requires particular attention, as the reuse of such treated wastewater effluents might pose a potential risk to the public health if disseminated into the environment during reuse events (4). As a first step to assess the risk associated with wastewater reuse, it is necessary to understand the differential fates and persistence of ARB and ARGs in the main biological treatment unit of anaerobic and aerobic wastewater treatment systems. Few studies comparing differential aerobic/anaerobic ARG removal have been performed and showed conflicting information. Molecular studies by Diehl and LaPara (9) and Burch et al. (10) found that ARGs tend to be removed more efficiently under anaerobic conditions. However, other studies suggest that combined anaerobic-aerobic (11) or, in contrast, completely aerobic conditions are more efficient at removing ARGs and ARB from wastewater (12).

Although informative, these earlier studies relied purely on molecular-based detection and did not examine the factors potentially shaping the decay or persistence of the ARG and the ARB host in the sludge. The formation of specialized persister cells is a strategy adopted by different types of bacteria, such as E. coli, to endure harsh environmental conditions (13). These cells are dormant variations of vegetative cells that occur at low frequencies within the bacterial population and are commonly known for their capacity to withstand supralethal concentrations of antibiotics (13–15). The contribution of persisters to the establishment of chronic infections due to several pathogens is well documented (16–18). However, little is known about the contribution of persisters to ARB survival in aerobic and anaerobic sludge. The earlier studies also did not examine the factors shaping the decay or persistence of the ARGs associated with the antibiotic-resistant host in anaerobic and aerobic sludge. These factors include the stability and persistence of ARGs as extracellular DNA and the potential for horizontal gene transfer (HGT) events when the ARG is encoded on a conjugative plasmid.

In this study, Escherichia coli strain PI7 was used as a model bacterium to examine the existing knowledge gaps associated with the fate and persistence of ARB and ARGs in anaerobic and aerobic sludge. This bacterium was previously isolated from wastewater and carries an extensive repertoire of ARGs, including a copy of *bla*_NDM-1_, in a plasmid of the IncF family identified earlier as pKOX_NDM1 (19). In the last decade, *bla*_NDM-1_ has undergone a pandemic spread among clinically relevant bacteria (20, 21). It has also been detected at alarming levels in different environmental compartments, including water (22, 23), soil (24), and wastewater (8, 19). Given the importance of wastewater in the environmental mobilization of ARB and ARGs, we aimed to evaluate the fate and persistence of our model bacterium E. coli PI7 and its associated IncF plasmid in anaerobic and aerobic sludge microcosms under various trace antibiotic concentrations. Using molecular tools, this study evaluated plasmid stability, host persistence, and HGT events under anaerobic and aerobic conditions. Chromosomal and plasmidic decay rates were measured by quantitative PCR (qPCR) coupled with propidium monoazide (PMA) to discriminate between dead cells or extracellular DNA and cells with intact cell membranes. In addition, the potential stability of extracellular DNA in the liquid fraction of anaerobic and aerobic sludge was also evaluated.

## RESULTS

### Differential decay of *bla*_NDM-1_ in anaerobic and aerobic sludge microcosms.

**FIG 1:**
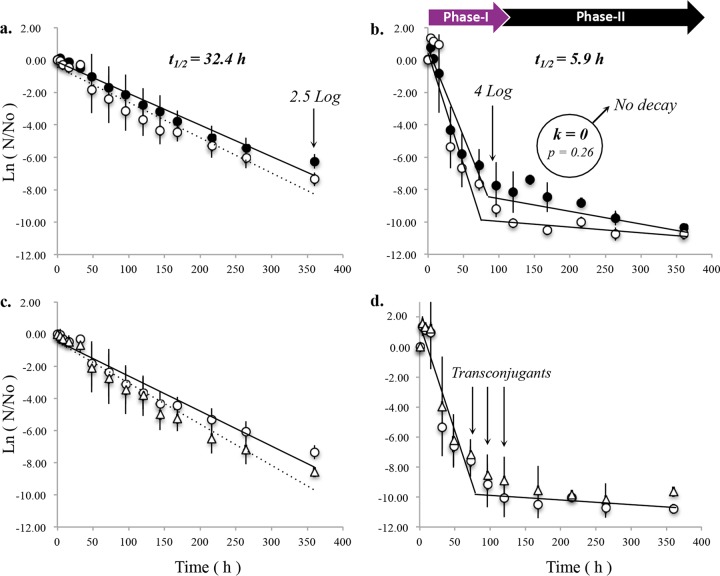
*bla*_NDM-1_ decay kinetics in nontreated (black circles) and PMA-treated (white circles) biomass fractions under anaerobic (a) and aerobic (b) conditions at 0 μg/liter of meropenem (*n* = 3). Plasmid stability under anaerobic (c) and aerobic (d) conditions was evaluated by comparing the *bla*_NDM-1_ (white circles) and *uidA* (white triangles) decay rates in PMA-treated biomass fractions (*n* = 3). Arrows in panel d indicate sampling points at which transconjugants were recovered by plating techniques.

Under anaerobic conditions, *bla*_NDM-1_ decayed following a first-order decay kinetic model (*R*^2^ > 0.935) (Fig. 1a). After 360 h of operation, *bla*_NDM-1_ copy numbers decreased by 2.5 log from 10^8^ to 10^6^ copies/g biomass. This decrease in the *bla*_NDM-1_ copy numbers corresponded to average first-order decay rates of −0.021 ± 0.002 h^−1^ (*t*_1/2_ = 32.9 ± 2.8 h) and −0.021 ± 0.001 h^−1^ (*t*_1/2_ = 32.3 ± 1.7 h) for the nontreated biomass (NTB) and PMA-treated biomass (PTB) fractions, respectively (Fig. 1a, Table 1).

**TABLE 1:** Decay rates of *bla*_NDM-1_ in PMA-treated biomass fractions of anaerobic and aerobic sludge microcosms under different trace meropenem concentrations

Microcosm	Replicate run	Meropenem concentration (μg/liter)^*a*^	*k* (h^−1^)^*b*^	*t*_1/2_ (h)	*P* value^*c*^
Anaerobic sludge	1	Control	−0.0202	34.3	
		100	−0.0223	31.1	0.33
	2	Control	−0.0219	31.7	
		100	−0.0213	32.5	0.15
	3	Control	−0.0223	31.1	NA
		100	−0.0205	33.8	0.98
Aerobic sludge	1	Control	−0.1361	5.1	
		1	−0.1460	4.7	0.77
		10	−0.1436	4.8	0.82
		100	−0.1386	5.0	0.94
	2	Control	−0.1107	6.3	
		1	−0.1095	6.3	0.70
		10	−0.0993	7.0	0.71
		100	−0.0923	7.5	0.46
	3	Control	−0.1119	6.2	
		1	−0.1253	5.5	0.49
		10	−0.1095	6.3	0.85
		100	−0.1133	6.1	0.96

a Control corresponds to 0 μg/liter of meropenem.

b *k* values for the aerobic microcosms correspond to phase I decay.

c *P* values correspond to the comparison of each decay curve derived from meropenem-spiked microcosms (i.e., 1, 10, and 100 μg/liter meropenem), with the decay model obtained in the respective control microcosms (0 μg/liter). No significant differences were observed in their *bla*_NDM-1_ decay at the different meropenem concentrations tested. *P* value corresponds to the testing of H_o_: *k*_control_ = *k_Xi_* μg/liter.

Under aerobic conditions, *bla*_NDM-1_ decay in the biomass fraction showed a biphasic behavior (Fig. 1b). An initial 4-log decrease in copy numbers from 10^8^ to 10^4^ copies/g biomass during the first 72 h of reactor operation was observed. Phase I fits a first-order decay kinetic model (*R*^2^ > 0.83), with average decay rates of −0.1049 ± 0.019 h^−1^ (*t*_1/2_ = 6.6 ± 1.2 h) and −0.1196 ± 0.014 h^−1^ (*t*_1/2_ = 5.8 ± 0.7 h) (Fig. 1b, Table 1) for the NTB and PTB fractions, respectively. Similar to results observed under anaerobic conditions, differences in the *bla*_NDM-1_ decay rates of the two fractions were not statistically significant (*P* = 0.92). After 72 h of aerobic exposure, decay was then followed by a plateau in phase II, with NTB and PTB average decay rates of −0.007 ± 0.005 h^−1^ (*R*^2^ = 0.917) and −0.005 ± 0.012 h^−1^ (*R*^2^ = 0.917), respectively. No statistically supported differences were observed between NTB and PTB decay rates, suggesting that most of the detected *bla*_NDM-1_ is harbored within cells with intact cell membranes. PTB decay rates (associated with cells with intact membranes) were statistically undistinguishable from a decay of zero (*P* = 0.26). Instead, *bla*_NDM-1_ abundance stabilized at 10^5^ copies/g biomass.

### Decay of *bla*_NDM-1_ correlates with E. coli PI7 chromosomal decay.

**FIG 2:**
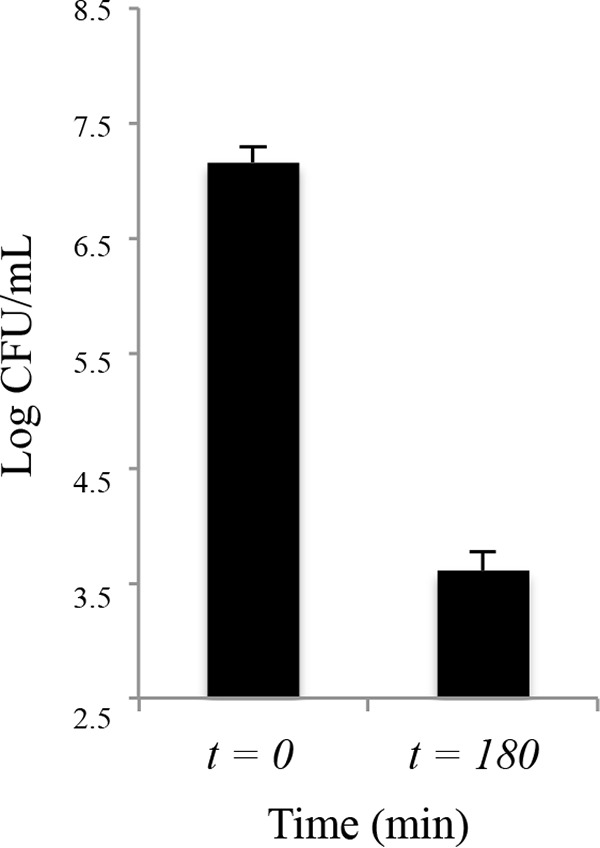
E. coli PI7 cell count before (*t* = 0 min) and after (*t* = 180 min) meropenem challenge at 640 μg/ml (*n* = 4). The persister cell frequency is expressed as the ratio of the cell count at *t* = 180 min and the cell count at *t* = 0 min, which corresponded to a frequency of 3.14 × 10^−4^ ± 1.5 × 10^−4^ (*n* = 4).

Due to the plasmidic origin of *bla*_NDM-1_, the decay kinetics of this ARG might be explained by (i) plasmid loss or (ii) cellular decay of the bacterial host. To determine which of these two factors mainly explained the *bla*_NDM-1_ decay observed in the microcosms, we compared the PTB decay rates of *bla*_NDM-1_ (indicative of plasmid decay rates) with the PTB decay rates of a chromosome-carried gene (*uidA*). In the anaerobic microcosms, the two target genes decayed at the same rate (*P* = 0.32) (Fig. 1c). Similarly, under aerobic conditions, phase I and plateau *bla*_NDM-1_ and *uidA* decay rates were statistically indistinguishable among all tested trace antibiotic concentrations (0.19 > *P* > 0.50) (Fig. 1d). *uidA* was initially spiked in the microcosm experiments at a frequency of 10^9^ copies/g biomass and stabilized at 10^5^ copies/g biomass during the plateau phase. These results suggest the presence of a recalcitrant subpopulation of E. coli cells, occurring at a frequency of 10^−4^ (plateau *uidA* copies/initial *uidA* spiking). The occurring frequency of recalcitrant *uidA* copies coincides with the frequency of persister cells of E. coli PI7 in pure culture (Fig. 2).

### Trace antibiotic concentrations do not influence the persistence of E. coli PI7 or its associated ARGs.

The results from the previous section suggest that *bla*_NDM-1_ decay is mainly explained by the cellular decay of the E. coli PI7 host. As residual antibiotics are commonly found in wastewater (25), there is a latent possibility that such trace antibiotic concentrations might provide a selective advantage to E. coli PI7 in the sludge, and consequently prolong the persistence of *bla*_NDM-1_. To evaluate this possibility, the decay of *bla*_NDM-1_ and *uidA* was assessed in the anaerobic microcosms at 0 and 100 μg/liter of meropenem and at 0, 1, 10, and 100 μg/liter of meropenem in the aerobic microcosms. None of the selected meropenem concentrations influenced *bla*_NDM-1_ or *uidA* decay kinetics under either anaerobic or aerobic conditions (see Fig. S1 and S2 in the supplemental material). NTB and PTB decay rates (Table 1) at the different antibiotic concentrations were also not statistically different from their respective controls (i.e., reactors without antibiotic; *P* > 0.15).

### Activated sludge as a reservoir for ARGs upon E. coli PI7 decay.

Due to the plasmidic origin of *bla*_NDM-1_, the sludge biomass might act as a sink for this gene through plasmid conjugation. Accordingly, HGT was assessed by culture-based methods. After 72 h of operation, transconjugants were recovered from the aerobic microcosm at three sampling points (Fig. 1d) at an average frequency of 10^3^ to 10^4^ CFU/g of biomass (see Text SI-1 in the supplemental material). Subsequent Sanger-based sequencing revealed that the transconjugants were *bla*_NDM-1_-positive isolates of Enterobacteriaceae belonging to Shigella and Citrobacter genera. No HGT events were detected by culture-dependent methods in the anaerobic microcosms.

### Differential decay of colloidal DNA between anaerobic and aerobic liquid fractions.

**FIG 3:**
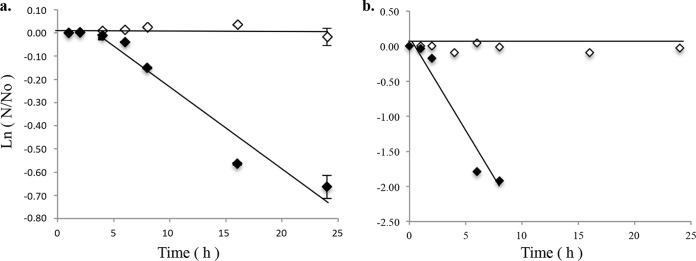
Decay of extracellular colloidal DNA in anaerobic (white diamonds) and aerobic (black diamonds) sludge liquid fractions measured by qPCR (a) and electroporation (b) assays.

Upon E. coli PI7 cellular decay, *bla*_NDM-1_ was detected in the supernatant fraction of the microcosm experiments by endpoint PCR (data not shown). To evaluate the potential persistence of extracellular DNA in liquid fractions of anaerobic and aerobic sludge fractions, a separate experiment was carried out. Naked plasmidic DNA was spiked in a dialysis cassette and its decay was tracked by qPCR and electroporation assays in TOP10 E. coli cells. As sequence-dependent DNA flexibility determines the cleave rate mediated by DNase I (26), the decay rate of the cloning vector does not necessarily represent the decay rate of the *bla*_NDM-1_ plasmid. Specifically, given the larger size of the *bla*_NDM-1_ plasmid (ca. 110 kbp), it may be more inclined to degrade faster than the smaller clone vector. Although the substitution of the clone vector may overestimate the persistence of the actual plasmid decay, this experiment is informative about the persistence potential of DNA/ARGs in anaerobic and aerobic liquid fractions. No decay was detected by qPCR or electroporation assays for plasmids spiked into the anaerobic liquid fraction (*P* = 0.5) (Fig. 3a and b). In contrast, in the aerobic liquid fraction, plasmid decay was detected by both qPCR and electroporation assays at rates of −0.036 ± 0.005 h^−1^ (*t*_1/2_ *=* 19.4 ± 2.8 h) and −0.278 ± 0.03 h^−1^ (*t*_1/2_ = 2.5 ± 0.25 h), respectively. Decay rates estimated by electroporation assays in the aerobic liquid fraction were significantly higher than those quantified by qPCR (*P* ≪ 0.05).

## DISCUSSION

The removal of ARB from wastewater, particularly strains that are resistant to new classes of antibiotics, is required to protect public health during reuse events (4). Both aerobic- and anaerobic-based wastewater treatment systems are utilized for the treatment of municipal wastewater. However, a key parameter to consider when implementing these systems is the decay rates of ARB and ARGs in the associated sludge and in the treated wastewater effluents.

Using *bla*_NDM-1_-positive E. coli PI7 isolated from wastewater as a model bacterium, it was observed that the decay rate of this bacterium was 1 order of magnitude higher in the aerobic sludge than in the anaerobic sludge microcosms. The longer persistence of E. coli PI7 observed under the anaerobic condition coincides with earlier microcosm studies comparing the differential anaerobic/aerobic survival of E. coli in diluted fermenter sludge (27), manure, manure slurry (28, 29), drinking water (30), and activated sludge (31). These studies provide evidence that oxygen is a key factor driving decay of E. coli in the secondary habitat, and partly explain the longer persistence of E. coli PI7 in anaerobic microcosms. Endogenous reactive oxygen species (ROS) are formed as byproducts during aerobic metabolism (32). ROS are highly oxidative molecules that can react with cellular components and lead to cytotoxic (32–34) and mutagenic defects in the cells (35). Several detoxification mechanisms have evolved in aerobic and facultative organisms in order to cope with the deleterious effects of ROS (32, 36). Even though E. coli possesses several of those detoxification mechanisms (32), it is possible that the cells experiencing oxidative stress undergo a decrease in fitness and hence decayed faster in the aerobic microcosms.

However, the presence of oxygen is most likely not the only factor driving the decay of E. coli PI7 in the microcosms, since decay was also observed in the anaerobic microcosms. In addition to ROS, the microbial community has been reported to play an important role in the decay of E. coli within the environment (37). The longer persistence of E. coli PI7 could also be partially explained by the fact that microbial communities in the gut and the anaerobic sludge tend to be more similar to each other, as exemplified by the predominance of Firmicutes and Bacteroidetes in both ecosystems (38), than are the gut microbiota and aerobic sludge (39–41). Due to these similarities in the microbial communities, E. coli PI7 might have acclimated better and decayed more slowly in the anaerobic sludge than aerobic sludge.

It was further observed that the decay kinetics of E. coli PI7 followed a biphasic decay behavior in the aerobic microcosms. This biphasic decay behavior has been documented extensively for E. coli and other fecal indicator bacteria (42, 43). After the initial spiking event, *bla*_NDM-1_ and *uidA* were detected in the aerobic reactors at an average frequency of 10^9^ copies/g biomass. After 72 h, no further decay was observed, and both gene copy numbers plateaued at an approximate frequency of 10^5^ copies/g biomass (Fig. 1b). As only one copy of *bla*_NDM-1_ or *uidA* is present per E. coli PI7 cell, these results indicate that 1 in 10,000 cells may be recalcitrant to decay (see Text SI-2 in the supplemental material). Previous studies on pure cultures of E. coli suggest this pattern resulted from heterogeneity within the bacterial population (44). We further hypothesized that the bacterial subpopulation responsible for the plateau phase phenotype corresponds to persister cells, a phenotypical variant of vegetative cells that exhibit improved tolerance to antimicrobials and other stressful environmental conditions (13). Indeed, the same E. coli PI7 was observed to upregulate transcriptional responses of genes related to persister cell formation upon solar irradiation (45), suggesting that E. coli PI7 adopts this strategy to facilitate its persistence during stressful conditions. Persisters typically occur at a fixed frequency between 10^−4^ and 10^−6^, depending on the E. coli strain (14). Consequently, in all aerobic microcosms (*n* = 12), the E. coli PI7 populations consistently stabilized at a frequency of 10^5^ cells/g biomass after a 4-log cell density decline (Fig. 1). A further evaluation of the frequency of persister cell formation in pure cultures of E. coli PI7 revealed that for every 10,000 vegetative cells, 1 persister cell is formed (frequency of 10^−4^) (Fig. 2), coinciding with the persisting cell numbers obtained in the decay experiments. Further evidence supporting the presence of persister cells in the aerobic sludge is the fact that even though *uidA* was consistently detected in PTB fractions at a frequency of 10^5^ copies/g sludge until the end of the decay experiments, E. coli PI7 was no longer recovered from the sludge by culture-based methods after 96 h of microcosm establishment (data not shown). These data suggest that E. coli PI7 possibly transitioned to a viable but nonculturable (VBNC) state that is consistent with the persister cell hypothesis.

Persister cells had been studied in clinical settings, as this dormant state allows antibiotic-susceptible bacterial populations to survive antimicrobial treatments (14). To the best of our knowledge, there is only one report of environmental incidence of a dormant but infective state of the fish pathogen Pasteurella piscicida (46). However, the importance of these specialized cells for the survival and dissemination of E. coli in the environment, particularly in the wastewater treatment system, has been overlooked (47). The occurrence of persisters of pathogenic strains in the sludge (such as E. coli PI7) has implications for the management and disposal of the sludge originating from WWTPs and their respective treated effluents. As this subpopulation of bacteria exhibits high tolerance to antimicrobials, this raises the question of whether our current disinfection practices are effective at fully inactivating persisters of pathogenic bacteria that remain in the treated wastewater. This can raise potential concerns when treated wastewater is intended for reuse, as improper inactivation might represent a direct risk to the public health.

An earlier study in reactors treating wastewater suggested that as a general trend, ARGs are removed more efficiently under anaerobic conditions (9). However, in a study by Diehl and LaPara (9), the anaerobic reactors were operated at a longer retention time than the aerobic reactors. This difference in the operational parameters of the systems makes it difficult to determine whether the improved ARG removal corresponded to factors related to the anaerobic/aerobic condition or to differences in the retention times of the two types of systems. Nonetheless, it is important to highlight that Diehl and LaPara (9) observed that some particular ARGs showed prolonged persistence under the anaerobic condition, suggesting that the differential ARG decay is dependent on each particular ARG. In agreement with this observation, Burch and collaborators (10) concluded that the removal rates of ARGs vary substantially depending on the specific ARG. In a more detailed study, Rysz and collaborators (48) indicated that the effect of oxygen availability on the maintenance of tetracycline resistance genes is also dependent on the host cell or, more specifically, on the particular plasmid-host pair. Anaerobic conditions led to complete loss of a plasmid carrying the tetracycline resistance gene (the RP1 plasmid) in Pseudomonas aeruginosa, while E. coli retained its tetracycline resistance plasmid (pSC101) for more than 500 generations. The pSC101 and *bla*_NDM-1_ plasmids harbored by E. coli PI7 (pKOX_NDM-1) carry a toxin-antitoxin system that might improve their retention in the E. coli host. Moreover, these two plasmids belong to the IncF family, which shows high stability in Enterobacteriaceae (49–52). These two characteristics shared by pSC101 and pKOX_NDM-1 might have accounted for their retention under energy-deprived anaerobic conditions. As the particular interaction of pKOX_NDM-1 and E. coli PI7 is highly stable, cellular decay of E. coli PI7 is the main factor driving the decay kinetics of *bla*_NDM-1_ in our microcosm experiments.

In a complex microbial community where competition for resources takes place (42), we expected that the addition of meropenem would have resulted in increased fitness of E. coli PI7 in the anaerobic and aerobic sludge. On the contrary, such antibiotic concentrations did not provide an evident selective advantage to E. coli PI7 that resulted in prolonged persistence. It is important to highlight that *bla*_NDM-1_ is fully functional in E. coli PI7, with an MIC of 64 μg/ml (19), suggesting that the possible adaptive advantage imposed by the meropenem addition might be negligible compared to the other negative pressures experienced by E. coli in the sludge environment.

Although we did not detect any effects of trace antibiotic concentrations on the survival of E. coli PI7 or on *bla*_NDM-1_ persistence, other studies suggested that exposure to sublethal antibiotic and disinfectant concentrations stimulate plasmid conjugation rates in activated sludge (53) and in water (54). Indeed, transconjugants were recovered from the aerobic microcosms. As IncF plasmids are unique to Enterobacteriaceae (55), all transconjugants isolated fall within this taxonomical unit, which includes many genera associated with waterborne pathogens, such as Salmonella, Yersinia, Klebsiella, Shigella, Citrobacter, etc. (56, 57). In contrast, no transconjugants were recovered from the anaerobic microcosms, most likely because such HGT events took place with fastidious bacteria highly prevalent in anaerobic environments (58), and these bacteria cannot be easily cultivated. Similarly, after 120 h of aerobic microcosm establishment, transconjugants were no longer recovered from the aerobic sludge (Fig. 1d). Lower plasmid stability of the new host-plasmid interaction is a plausible explanation for the loss of transconjugant recovery. However, IncF plasmids are highly stable in the Enterobacteriaceae (49–52), and such stability is likely improved by the toxin-antitoxin system (19). Due to the phylogenetic proximity of Shigella and Citrobacter spp. to E. coli (59), it is speculated that this plasmid might also be stable in these hosts. Hence, an alternative explanation for the lack of transconjugant recovery is the loss of culturability of such bacteria, since the progression to a viable but nonculturable (VBNC) state is a survival strategy common among diverse groups of bacteria (60).

The contribution of HGT and the factors affecting the dissemination of plasmids in activated sludge under laboratory conditions have been well documented. These factors include plasmid host range (61), sludge retention times (62), stressful environmental conditions (53), and host/donor phylogenetic affiliations (63). Significant enrichment of the ratios of multiple ARGs compared to 16S rRNA genes throughout the wastewater treatment process confirms the potential for mobility and proliferation of ARGs within the activated sludge microbial communities in full-scale treatment systems (64). *bla*_NDM-1_ HGT events involving activated sludge microorganisms and soil bacteria had also been reported (8). In agreement with these studies, our results emphasize the role of activated sludge as an environmental reservoir of plasmid-carried ARGs, as well as its potential role in mediating the dissemination of ARGs into the environment and to other Enterobacteriaceae.

Subsequently, upon cellular decay of E. coli PI7 and the previously identified transconjugants, *bla*_NDM-1_ can be released to the nonsettleable or colloidal fraction of the sludge. The plasmid DNA (pDNA) decay experiments showed that although the decay at the gene level is considerably slower (measured by qPCR), the fragmentation process of naked DNA in the liquid aerobic fraction leads to rapid decay in the replicative structure of the plasmid (measured by electroporation assays). Circular plasmids replicate by rolling-circle, strand displacement, or theta replication. In these three replication mechanisms, the circular structure of at least one DNA strand must be maintained in order to complete the replication cycle (65). A single double-strand break would linearize the plasmid, resulting in the disruption of the replication process by any of these mechanisms. Ultimately, the lack of replicative functions would compromise the ability of the plasmid to be disseminated and maintained in the transformed bacterial host.

DNA degradation in the environment is a complex multifactorial process involving chemical and biological aspects (66). DNase-mediated degradation is the main biological process driving the decay of extracellular DNA in the environment (67, 68), while low temperatures, high salinity, high levels of organic matter, and anoxic environments are some of the physicochemical factors that contribute to the preservation of environmental DNA (66, 69). Determining the factors influencing the persistence of DNA in sludge liquid fraction is outside the scope of this study. However, it was determined that DNA persisted for a longer time in the liquid fraction of the anaerobic sludge than in that of the aerobic sludge, ultimately increasing the probability of subsequent *bla*_NDM-1_ uptake and fixation by environmental bacteria. In a previous study, it was found that membranes of 100 kDa and smaller could achieve significant removal of ARGs, attaining up to 4.5-log removal of colloidal DNA (70). Hence, coupling membrane separation with the activated sludge process can serve to mitigate microbial risk associated with the presence of persisters and extracellular DNA in the treated effluent.

In summary, the results from this study highlight the higher potential of dissemination of E. coli PI7 and ARGs associated with prolonged host and extracellular DNA persistence in anaerobic sludge. In the aerobic sludge, this study demonstrates transconjugation of plasmids encoding ARGs to compatible bacteria within the sludge, highlighting the likelihood of potential horizontal gene transfer events. Furthermore, this study emphasizes the potential importance of persister cells in the survival and dissemination of enteric pathogens into the natural environment and suggests a certain extent of indirect and direct risk to public health imposed by the presence of persister cells of pathogenic strains remaining in the sludge and effluents of WWTPs.

## MATERIALS AND METHODS

### Microcosm preparation.

Two sets of microcosm experiments, representing anaerobic and aerobic biological reactors, were established in 1-liter sterile Pyrex bottles. Anaerobic and aerobic microcosms were seeded with 800 ml of sludge (mixed liquor suspended solids [MLSS] of 4 to 5 g/liter) from a lab scale anaerobic bioreactor (38) and an aerobic full-scale WWTP, respectively. The sludge in the anaerobic bioreactor was comprised of camel feces and anaerobic sludge from an industrial WWTP in Riyadh, and the anaerobic bioreactor had been in operation for more than 3 years (38, 71, 72). The full-scale WWTP was located at King Abdullah University of Science and Technology in Thuwal, Saudi Arabia, and had a capacity of 1,600 m^3^/day. Hydraulic retention time (HRT) and sludge retention time (SRT) in the activated sludge tank were 2.5 h and 40 days, respectively. The sludge tanks were operated at an average temperature of 33°C with 1 to 2 mg/liter of dissolved oxygen at pH 7 to 8. Sludge for each replicate run (*n* = 3) was recovered at three different time points scattered along a 1-year period for both anaerobic and aerobic seed sludge. Prior to the E. coli PI7 spiking event, the seed sludge was acclimated for 10 days and screened for the presence of *bla*_NDM-1_ or *uidA*. No *bla*_NDM-1_ or *uidA* copies were detected in anaerobic and aerobic sludge by either endpoint PCR or cultivation methods before E. coli PI7 spiking.

### Decay experiments.

The E. coli PI7 inoculum to be spiked into the microcosms was grown in LB broth at 37°C for 8 h to an optical density at 600 nm (OD_600_) of 0.7. Once this cell density was reached, 100 ml of culture were spiked into each microcosm, resulting in a final volume of 900 ml per microcosm. The addition of this large quantity of E. coli PI7 cells into each individual microcosm, albeit not representative of actual conditions in WWTPs, was similar to approaches undertaken in earlier studies (73, 74). The high cell density spiked into the microcosm was required to ensure final cell density upon decay remained within the limits of detection by qPCR, and also to allow detection of subpopulations (e.g., persister cells) that generally occur in low cell densities. Decay experiments were performed at different trace antibiotic concentrations. In the anaerobic microcosms, decay was evaluated at 0 and 100 μg/liter of meropenem (*n* = 2 per replicate run) (Sigma-Aldrich, St. Louis, MO, USA). In the aerobic microcosm, concentrations of 0, 1, 10, and 100 μg/liter of meropenem were tested (*n* = 4 per replicate run). Anaerobic microcosms were not tested with 1 and 10 μg/liter meropenem because of low sludge production from the anaerobic bioreactor, and this restricted the number of microcosms that could be set up. Concentrations were chosen to represent the concentration range of organic micropollutants commonly reported in municipal wastewater (75, 76). Three independent replicate runs for each aerobic and anaerobic set-up were performed, comprising a total of 6 and 12 anaerobic and aerobic microcosms, respectively. Microcosms were operated as sequencing batch reactors at a constant temperature of 37°C. The aerobic microcosms were aerated with atmospheric air at 250 ml/min, while the anaerobic microcosms were established in airtight bottles and mechanically stirred at 250 rpm. To prevent oxygen intrusion, anaerobic microcosms were established and sampled in a vinyl anaerobic chamber (Coy Lab Products, MI). After each feeding/sampling event, anaerobic microcosms were purged with 99.9% nitrogen for 15 min. MLSS and pH were measured every 24 h for all microcosms, and pH was maintained at 7.2 ± 0.4 using HEPES buffer at a final concentration of 25 mM. Daily, 100 ml of liquid was removed from the aerobic and anaerobic microcosms and replaced with synthetic wastewater (38) that had the corresponding concentration of antibiotic, achieving a food-to-microorganism ratio (F/M) of 0.2. Biomass was separated from the nonsettleable liquid fraction by centrifugation at 9,800 × *g* for 20 to 30 min in a sterile ultracentrifuge bottle, and reintroduced into the respective microcosm.

### Sample processing.

At each sampling event, 14 ml of sludge was collected from each microcosm and a subset was used for (i) total biomass DNA isolation (2 ml), (ii) sludge exposure to propidium monoazide (PMA) (0.5 ml), and (iii) detection of horizontal gene transfer (HGT) events by culture-based methods (0.5 ml). Sludge exposure to PMA was performed as previously described (77). Given that PMA can be limited in its accuracy to differentiate between cells with compromised and intact cell membranes, more details on PMA exposure protocol and validation are given in Text SI-3 and Table S1 in the supplemental material.

Sludge samples for total DNA isolation and PMA-treated samples were centrifuged at 10,000 × *g* for 5 min and the supernatant fraction was discarded. The remaining biomass pellet was immediately frozen at −80°C, and subsequently lyophilized using the Alpha 1-2 LDplus freeze dryer (Martin Christ GmbH, Germany). After completion of the drying cycle, dry biomass weights were recorded and samples were ready for DNA extraction.

### Horizontal gene transfer detection by culture-based methods.

To detect potential conjugation events, the remaining 0.5 ml of the initial 14 ml sludge sample were serially diluted and plated on MUG-EC medium (Sigma-Aldrich, St. Louis, MO) with 1.5% (wt/vol) agar and 8 μg/ml meropenem. MUG-EC allows the rapid screening of E. coli isolates, as MUG (methylumbelliferyl-β-glucuronide) cleavage by the glucuronidase enzyme leads to the formation of colonies that exhibit blue fluorescence under UV (78). Nonfluorescent colonies were selected as potential transconjugants, and subsequently confirmed by colony PCR using *bla*_NDM-1_-specific primers as shown in Table 2. Strain identity was determined by 16S rRNA gene sequencing using the 11F/1492R primer pair (79). The detection limit of culture-based methods performed in this study was determined to be 10^4^ CFU/g sludge (see Text SI-4 in the supplemental material).

**TABLE 2:** Primers and probes used in this study

Primer or probe	Gene target	Amplicon size (bp)	Sequence^*a*^	Cycling conditions	Use
Primers					
NDM154-F^*b*^	*bla*_NDM-1_	154	ATTAGCCGCTGCATTGAT	50°C × 2 min; 95°C × 20 s; 40 cycles of 95°C × 1 s and 60°C × 20 s	qPCR
NDM154-R^*b*^			CATGTCGAGATAGGAAGTG		
uidA159-F^*c*^	*uidA*	159	CGAATCCTTTGCCACGCAAG	50°C × 2 min; 95°C × 20 s; 40 cycles of 95°C × 1 s and 60°C × 20 s	qPCR
uidA159-R^*c*^			TCACAGCCAAAAGCCAGACA		
NDM640-F	*bla*_NDM-1_	640	TAGTGCTCAGTGTCG	95°C × 3 min; 35 cycles of 95°C × 30 s, 60°C × 30 s and 72°C × 1 min; final elongation 72°C × 5 min	Sequencing (transconjugant screening)
NDM640-R			CATTAGCCGCTGCA		
11F	16S	1481	GTTYGATYCTGGCTCAG	95°C × 5 min; 35 cycles of 95°C × 1 min, 45°C × 45 s and 72°C × 2 min; final elongation 72°C × 10 min	Sequencing (transconjugant identity)
1492R			GGYTACCTTGTTACGACTT		
Probes					
NDM-22^*b*^	*bla*_NDM_		56-FAM/AGACATTCG/ZEN/GTGCGAGCTGGCGGA/3IABkFQ	As described for primer pair	qPCR
uidA-23^*c*^	*uidA*		56-FAM/TCGCCCTTC/ZEN/ACTGCCACTGACCG/3IABkFQ	As described for primer pair	qPCR

a FAM, 6-carboxyfluorescein.

b Primer-probe pair NDM-154 and NDM-22 qPCR amplification efficiency was 98%.

c Primer-pair uidA159 and uidA-23 qPCR amplification efficiency was 102%.

### Colloidal DNA decay.

E. coli PI7 carries a 110-kbp plasmid encoding NDM-1 (19). Plasmid integrity was determined by electroporation into Invitrogen TOP10 electrocompetent cells (Thermo Fisher Scientific, Carlsbad, CA, USA). Due to the low electroporation frequencies of this large plasmid, decay experiments were carried out using the pCR2.1 cloning vector (Thermo Fisher Scientific, Carlsbad, CA, USA) that harbors a 640-bp *bla*_NDM-1_ gene insertion (i.e., a total plasmid vector size of ∼4.6 kbp). Decay experiments for the plasmid were performed in the liquid fraction of either aerobic or anaerobic sludge collected from a local WWTP and a lab scale anaerobic membrane bioreactor (AnMBR), respectively. The sludge liquid fraction was separated from the biomass by centrifugation at 10,000 × g for 20 min. Supernatant was recovered and filtered with cheesecloth to further remove biomass in suspension. Subsequently, 1.6 ml of plasmidic DNA (10^10^ copies/μl) were dosed into a Float-A-Lyzer G2 dialysis device with a molecular mass cutoff of 100 kDa (Spectrum Laboratories, Rancho Dominguez, CA), and the device was submerged in the liquid fraction of either aerobic or anaerobic sludge for a period of 24 h. Anaerobic decay experiments were performed under anaerobic conditions in a vinyl anaerobic chamber with an atmosphere of 95% nitrogen and 5% hydrogen (Coy Lab Products, MI, USA). A total of 50 μl samples were taken from the dialysis device at each sampling point, and used for (i) plasmidic DNA quantification with qPCR and (ii) plasmid integrity quantification by electroporation in TOP10 cells (see Text SI-5 in the supplemental material).

### DNA extraction.

Lyophilized PMA-treated sludge and non-PMA-treated sludge fractions were subjected to DNA extraction using the PowerSoil DNA extraction kit (Mo Bio) with slight modifications to the manufacturer's protocol, as previously described (4).

### Gene quantification and statistical tests.

The *uidA* and *bla*_NDM-1_ genes were used as the chromosomal and plasmidic markers, respectively. β-d-Glucuronidase (*uidA*) was selected as the chromosomal marker since only one copy of this gene is present per E. coli genome (19). Differences in the decay of the chromosomal and plasmidic material of E. coli PI7 were therefore assessed by comparing PMA-treated biomass (PTB) decay rates of *uidA* and *bla*_NDM-1_ genes, respectively. *bla*_NDM-1_ and *uidA* copy numbers were determined by absolute quantification on an Applied Biosystems 7900HT Fast real-time PCR system (Thermo Fisher Scientific, Carlsbad, CA, USA). PCR primers and TaqMan probe sequences are listed in Table 2. MLSS measurements were fairly stable in the microcosm experiments during the whole length of the experiment (see Fig. S3 in the supplemental material), suggesting stability of both aerobic and anaerobic microbial communities in the microcosms. Gene copy numbers were normalized by dry weight of biomass. Decay rates were expressed as ln(*N*/*N*_o_), where *N* corresponds to the copy numbers at *t* = *X_i_*, and *N*_o_ corresponds to the copy numbers at *t* = 0. Half-life was calculated using a first-order decay kinetic model. Linear regressions were performed using the least-squares method and the significance of the slopes of the regression models (β ≠ 0) was evaluated using the *t* test. Decay curves were compared using the model *Y*_i_ = β_0_ + β_1_*X_i_*_1_ + β_2_*X_i_*_2_ + β_3_*X_i_*_1_*X_i_*_2_ (80). All statistical analysis was done using StatPlus at 95% confidence unless otherwise stated.

### Determination of persister cell frequency in pure cultures of E. coli PI7.

Persister cells are dormant variations of vegetative cells that can withstand harsh environmental conditions, including exposure to supralethal concentrations of antibiotics. This resistance to antibiotics is not encoded in the chromosome but is rather a consequence of their dormant phenotype. The frequency of persister cells in this study was determined using a modified protocol described by Keren et al. (15). Modifications were made to the type of antibiotic used and the working concentration. As E. coli PI7 exhibits extremely high tolerance to ampicillin, the antibiotic challenge was performed with meropenem, a carbapenem exhibiting a similar mode of action (7). E. coli PI7 was challenged with a supralethal meropenem concentration of 640 μg/ml, corresponding to a 10-fold increase of the MIC and a 5-fold increase of the lethal meropenem concentration reported for this strain (19). In summary, overnight cultures of E. coli PI7 were diluted 1:1,000 in LB broth without meropenem and incubated at 37°C and 200 rpm to a final OD_600_ of 0.2. To provide a baseline cell count (*t* = 0), 10 ml of culture from each flask (*n* = 4) were pooled and placed at 4°C. Subsequently, cell cultures were challenged with meropenem (640 μg/ml), and incubated at 37°C and 200 rpm for 180 min. Cell counts were determined by serial dilution and plating in MUG-EC agar plates. Persister cell frequency was expressed as the ratio of the cell count at *t* = 180 min to the cell count at *t* = 0.

## Supplementary Material

Supplemental material
