# Supplementary material for: Fate and Persistence of a Pathogenic NDM-1-Positive Escherichia coli Strain in Anaerobic and Aerobic Sludge Microcosms
Source: Appl Environ Microbiol. 2017 Jun 16;83(13):e00640-17. doi: 10.1128/AEM.00640-17 (PMC5479002; doi:10.1128/AEM.00640-17)
Supplement: Supplemental material [file supp_83_13_e00640-17__index.html]

Supplemental material 

# Fate and Persistence of a Pathogenic NDM-1-Positive Escherichia coli Strain in Anaerobic and Aerobic Sludge Microcosms

## Supplemental material

- Supplemental file 1 -

  Transconjugant calculations (Supplemental Information 1 [SI-1]); calculation of frequency of recalcitrant cells in microcosm experiments (SI-2); PMA exposure protocol and validation (SI-3); calculation of detection limit for culture-based methods (SI-4); plasmid integrity quantification by electroporation (SI-5); PMA validation on sludge samples (Table S1); *bla*NDM-1 decay curves for PMA-treated biomass samples from anaerobic sludge mesocosms at 0 and 100 μg of meropenem/liter (Fig. S1); *bla*NDM-1 decay curves for PMA-treated biomass samples from aerobic sludge mesocosms at 0, 1, 10, and 100 μg of meropenem/liter (Fig. S2); average MLSS for all replicate anaerobic and aerobic mesocosms (Fig. S3).

  PDF, 220K
