# Supplementary material for: Fate and Persistence of a Pathogenic NDM-1-Positive Escherichia coli Strain in Anaerobic and Aerobic Sludge Microcosms
Source: Appl Environ Microbiol. 2017 Jun 16;83(13):e00640-17. doi: 10.1128/AEM.00640-17 (PMC5479002; doi:10.1128/AEM.00640-17)
Supplement: Supplemental material [file AEM.00640-17_zam999117893s1.pdf]

Supplementary information for

Fate and persistence of a pathogenic NDM-1-positive *Escherichia coli* strain in anaerobic and aerobic sludge microcosms

David Mantilla-Calderon, and Pei-Ying Hong*

1. Transconjugant calculation in CFU per gram. 100 µL of diluted or undiluted sludge was plated onto MUG-EC agar plates supplemented with meropenem (8 µg/mL). Using the MLSS data (g/mL or g/L) the incidence of transconjugants in CFU/g was calculated as follow.

$$Incidence\ \left({}^{CFU}/_{g}\right) = incidence\ \frac{CFU}{mL} * \frac{1}{MLSS({}^{g}/_{mL})} = Incidence\ \frac{CFU}{g}$$

2. Frequency of recalcitrant cells in microcosm experiments. *uidA* was detected at a density of 10^9 copies/g of sludge immediately after the spiking of *E. coli* PI7, resulting in an initial cell density of 10^9 *E. coli* PI7 cells per g of sludge biomass. The frequency of recalcitrant cells was therefore calculated based on the following formula:

$$Frequency\ of\ recalcitrant\ cells = \frac{uidA\ copy\ \#\ in\ plateau\ phase}{uidA\ copy\ \#\ spiked} = \frac{10^5\ \frac{copies}{g}}{10^9 \frac{copies}{g}} = 1 * 10^{-4}\ or\ \frac{1\ uid\ recalcitrant\ copy}{10^4\ uidA\ copies}$$

3. PMA exposure protocol and validation. PMA is a high-affinity photo-reactive DNA binding dye, which upon light exposure covalently binds to the DNA molecule and inhibits downstream PCR amplification. The dye is impermeable to cell membranes, and would only bind to intracellular gene targets when cell walls and membranes are compromised. [1]. In activated sludge, PMA could be absorbed onto the suspended solids, compromising

its ability to intercalate with extracellular DNA and inhibit downstream PCR detection. To account for this, we used the PMA exposure protocol optimized by Bae and coworkers [2], and validated it in the sludge fraction used in this study.

E. coli PI7 was grown in LB with 16 µg/mL meropenem at 37 °C to an OD_{600} of 0.7. Cells were washed twice with 1X PBS, and resuspended in 30% of the original culture volume. Washed cells were dispensed in 1 mL aliquots, and half of the aliquots were heat-lysed at 95 °C for 30 min in a heat block. Subsequently, 200 µL of heat-lysed and 200 µL of non-lysed *E. coli* cells were added to a 15 mL centrifuge tube containing 2 mL of diluted activated sludge or anaerobic sludge to a mixed-liquor suspended solids concentration of 1 g/L. Each of the spiked sludge samples was divided into 2 fractions of equal volume, and transferred to transparent 2 mL eppendorf tubes. One of the portions was treated with PMA (Biotium) to a final concentration of 100 µM, and exposed to blue
L.E.D light for 10 min using a PMA-Lite Photolysis device (Biotium), while the other fraction remained untreated for PMA. PMA-treated and non-treated samples were frozen at -80 °C and lyophilized using a Christ Alpha 1-2 LD Plus freeze Dry.

Table S1 shows the results of the bla_{NDM-1} qPCR quantification in PMA-treated and non-treated sludge fractions. At these exposure conditions, PMA completely inhibited the PCR signal from the cells with uncompromised cell membranes. As Bae and coworkers described, PMA permeation in cells with uncompromised membranes was also observed, accounting for a 13-15% penetration in cells with uncompromised cell membranes. Although penetration in cells with uncompromised cell membranes occurs, inhibition of extracellular DNA or cells with compromised cell membranes is completely inhibited.

4. Calculation of detection limit for culture-based methods. 100 µL of diluted or non-diluted sludge were plated in MUG-EG agar plates supplemented with meropenem (8 µg/mL). The detection limit was established based on the minimum cell density (MCD) required to detect at least 10 CFU when plating 100 µL of non-diluted sludge. As the average MLSS corresponded to 4 g/L, each 100 µL aliquot contained 4 x 10^{-4} g of sludge biomass.

$$Sludge\ plated\ (g) = 4\,\frac{g}{L} * \frac{1L}{1000\ mL} * 0.1\ mL = 4 * 10^{-4}\ g$$

To obtain at least 10 CFU the minimum detectable cell density (MDCD) would correspond to 2.5 x 10^4 CFU/g

$$MDCD \left({}^{cfu}/_{g}\right) = \frac{10\ cfu}{4 * 10^{-4}\ g\ plated} = 2.5 * 10^{-4}\ cfu/g$$

5. Plasmid integrity quantification by electroporation. From the colloidal DNA decay experiment, 50 µL of DNA were recovered at each sampling point. As the DNA contained in the dialysis device was of extracellular nature, no extraction was performed.

Instead, recovered DNA was directly used for quantification by qPCR and electroporation assays. The cloning vector pCR®2.1 carry an ampicillin resistance gene, which was used for screening of transformants in TOP10. Electroporation was used to assess plasmid integrity, as only a fully functional plasmid molecule would lead to the ampicillin resistant phenotype in TOP10. Electroporation was performed using a Gene Pulser Xcell™ (Bio-Rad), in 2 mm electroporation cuvettes. Each electroporation reaction (2500 kV, 200 Ω and 25 µF) used 40 µL of electrocompetent TOP10 cells and 5 µL of plasmidic DNA from the colloidal decay experiments. Electroporated TOP10 cells were plated in LB plates containing 100 µg/mL of ampiciling, and the plates were incubated at 37 °C for 24 h. Transformants were enumerated and decay was expressed as Ln (N/N_o), where N_o corresponds to the number of transformants at $t = 0$ and N the number of transformants at $t = x$.

Table S1. PMA validation on sludge samples. Expected values are calculated based on a 50% reduction (0.3 Log) in the qPCR signals from the PMA negative fraction (total DNA fraction).

Replica	*Log PMA -*			*Log PMA +*			*Average*		*Expected*	*%error*
							PMA -	*PMA +*		
1	7.2	7.3	7.2	6.1	6.1	6.0	7.3	6.1	7.0	13
2	7.3	7.2	7.3	6.1	6.1	6.1	7.3	6.1	7.0	13
3	7.3	7.2	7.2	6.0	6.0	6.0	7.2	6.0	6.9	13
4	7.3	7.4	7.3	6.0	6.0	6.0	7.4	6.0	7.1	15

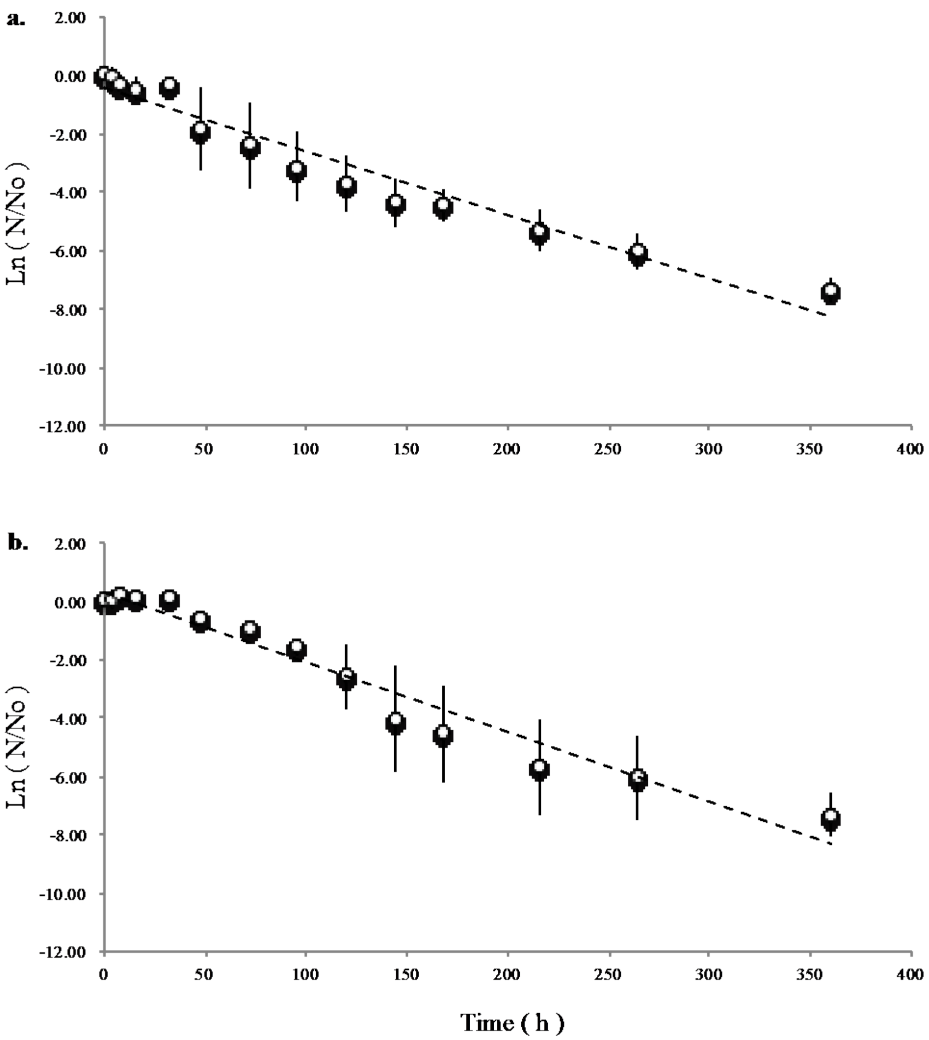

Figure S1. *bla*$_{NDM\text{-}1}$ decay curves in PMA-treated biomass samples from anaerobic sludge mesocosms at **(a)** 0 µg/L (n = 3) and **(b)** 100 µg/L of meropenem (n = 3). Plots comprise data from three independent replicate runs for each antibiotic condition.

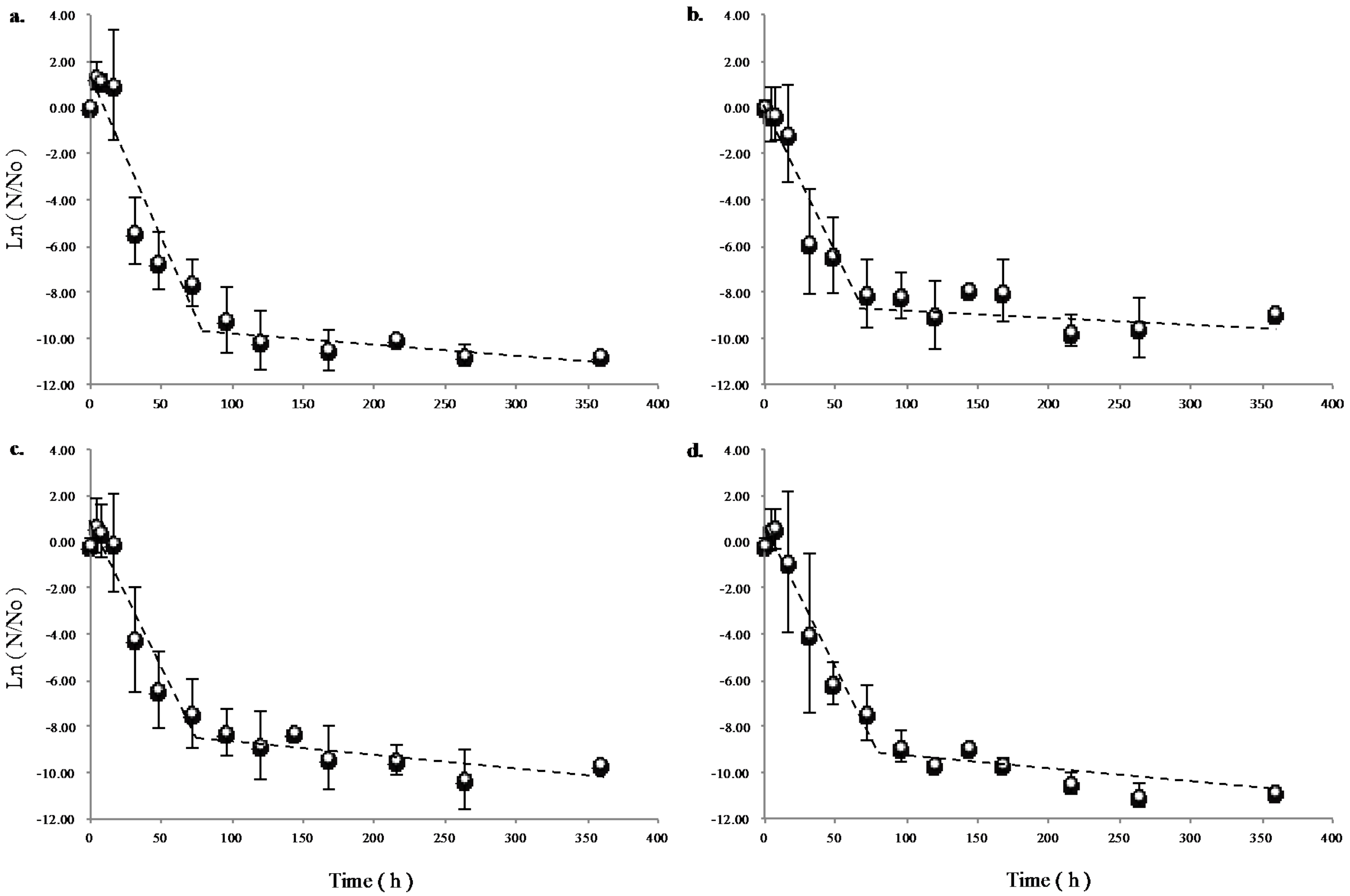

Figure S2. *bla*$_{NDM-1}$ decay curves in PMA-treated biomass samples from aerobic sludge mesocosms at **(a)** 0 µg/L, **(b)** 1 µg/L, **(c)** 10 µg/L and **(d)** 100 µg/L of meropenem. Plots comprise data from three independent replicate runs for each antibiotic condition (n = 3).

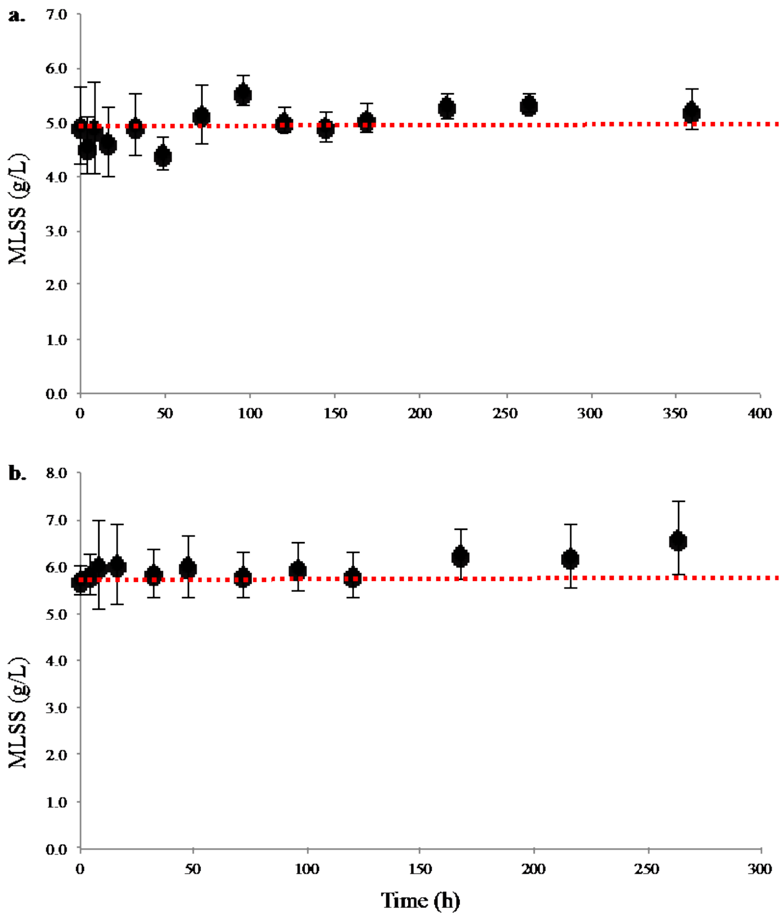

Figure S3. Average MLSS for all replicate **(a)** anaerobic mesocosms (n = 6) and **(b)** aerobic mesocosms (n = 12). The red dotted line represents the MLSS value at t = 0.

REFERENCES

1. **Nocker A., Cheung CY, Camper AK**. 2006. Comparison of propidium monoazide with ethidium monoazide for differentiation of live vs. dead bacteria by selective removal of DNA from dead cells. J Microbiol Methods **67**: 310-20.
2. **Bae S, Wuertz S.** 2009. Discrimination of viable and dead fecal Bacteroidales bacteria by quantitative PCR with propidium monoazide. Appl Environ Microbiol **75**: 2940-4.
3. **Al-Jassim N., et al.** (2015) Removal of bacterial contaminants and antibiotic resistance genes by conventional wastewater treatment processes in Saudi Arabia: Is the treated wastewater safe to reuse for agricultural irrigation? Water Res. **73**: 277-90.
